# Effects of multiple-intensity SSIT pairing patterns on explosive power and skill performance in female basketball players

**DOI:** 10.3389/fphys.2025.1635508

**Published:** 2025-09-01

**Authors:** Junsheng Cao, Jinyang Lin, Xiangying Wang, Yulong Wang

**Affiliations:** ^1^ School of Physical Education, Shandong Normal University, Jinan, Shandong, China; ^2^ Department of Physical Education, Central South University, Changsha, Hunan, China; ^3^ Yulin Campus, Guangxi Medical University, Yulin, Guangxi, China

**Keywords:** short sprint interval training (SSIT), female athletes, explosivepower, sprint performance, fatigue resistance, basketball conditioning, high-intensity training, agility

## Abstract

**Background:**

Explosive power and skill performance are critical components of basketball success, particularly in female athletes whose neuromuscular and physiological responses may differ from males. While Short Sprint Interval Training (SSIT) is recognized for improving aerobic and anaerobic capacity, its effect on explosive performance remains underexplored, especially across varied intensity pairings.

**Methods:**

A randomized controlled trial was conducted involving 36 female collegiate basketball players assigned to high-intensity (HI-SSIT), moderate-intensity (MI-SSIT), or multiple-intensity (MUL-SSIT) SSIT protocols over 8 weeks. Pre- and post-intervention assessments included vertical jump (CMJ, approach jump), sprint (10 m, 20 m), agility (Modified T-test, defensive slide), repeated sprint ability (RSA), intermittent endurance (YYIR1), and physiological markers (heart rate, blood lactate).

**Results:**

MUL-SSIT showed “possibly” beneficial effects on jump height decrement, sprint performance, and heart rate recovery compared to other protocols. While all groups improved in RSA and endurance capacity (p < 0.001), MUL-SSIT had the greatest gains in 10 m sprint and fatigue resistance. No significant improvements were observed in CMJ or agility across groups. Heart rate recovery improved in all protocols, with MUL-SSIT showing the most favorable outcomes.

**Conclusion:**

Multiple-intensity SSIT protocols are effective in enhancing fatigue resistance, sprint capacity, and certain aspects of explosive performance in female basketball players. These findings support the inclusion of varied-intensity SSIT formats in basketball conditioning programs to better address sport-specific demands.

## Introduction

Explosive performance is a critical factor in basketball performance, particularly given the sport’s dynamic and fast-paced nature (Stojanovic et al., 2012). The rapid generation of high forces is essential for executing skills that confer offensive and defensive advantages on the court (Erčulj et al., 2010). Basketball involves a unique blend of explosive physical demands and high-level technical skills, necessitating training strategies that enhance both aspects simultaneously (Castagna et al., 2009). With the increasing participation of female basketball players across various levels, there is a growing focus on optimizing explosive performance and skill performance through research and training practices. While traditional methods like resistance training and plyometric exercises have been commonly employed to explosive performance (Griffiths et al., 2019), these approaches, though effective, often entail lengthy training sessions and may not fully replicate the intermittent and high-intensity nature of actual game scenarios.

Short sprint interval training (SSIT) has recently gained prominence as an efficient and adaptable approach to enhancing aerobic and anaerobic capacities, particularly through the incorporation of diverse intensity levels. By mirroring the exertion-rest sequences characteristic of competitive events, SSIT fosters pronounced neuromuscular adaptations (Gibala et al., 2006; Burgomaster et al., 2005). Furthermore, the short duration of each sprint in SSIT minimizes the risk of overtraining and excessive fatigue, thereby enabling athletes to sustain quality efforts across training sessions. Key studies have shown that even a low volume of SSIT can lead to substantial improvements in metabolic and neuromuscular function (Gibala et al., 2006; Burgomaster et al., 2005). While early research mainly focused on endurance adaptations and metabolic efficiency enhancements, recent literature suggests that SSIT protocols can also induce neuromuscular changes that promote explosive performance. These adaptations include improvements in muscle buffering capacity, glycogen storage, and increased expression of oxidative enzymes, contributing to enhanced rate of force development and jump performance (Burgomaster et al., 2006). Consequently, SSIT presents a promising training approach not only for boosting overall cardiorespiratory fitness but also for augmenting explosive muscular performance.

In addition to the metabolic and neuromuscular benefits, SSIT protocols have been shown to induce significant changes in hormonal profiles, substrate utilization, and muscle fiber recruitment patterns—all of which are critical determinants of explosive performance. For instance, studies have reported that SSIT can modulate insulin sensitivity, enhance dopamine responsiveness, and even influence the activation of type II muscle fibers, which are primarily responsible for rapid and powerful movements (Richards et al., 2010). These adaptations are particularly important in female athletes who may exhibit different hormonal responses and muscle fiber compositions relative to their male counterparts (Zhang et al., 2023). Moreover, the incorporation of multiple-intensity pairing strategies may further optimize these adaptations by carefully balancing periods of high-intensity stress with recovery intervals that facilitate neuromuscular restitution and subsequent power output (Liu et al., 2024; Li and Xue, 2024). Thus, using multiple-intensity SSIT pairing patterns holds promise for significantly improving explosive performance while also addressing the technical demands of skill performance.

Thus, the aim of this study was to explore the effects of multiple-intensity SSIT pairing patterns on explosive performance and skill performance in female basketball players. We hypothesize that 8-week multiple-intensity SSIT can significantly improve explosive performance and skill performance in female basketball players.

## Methods

### Study design

A randomized controlled trial with a parallel group design was conducted to evaluate the effects of different Short Sprint Interval Training (SSIT) intensity distributions on explosive performance and skill performance in female basketball players. The study was approved by the Shandong Normal University Institutional Review Board (approval number: SDNUTYDW2024042), and all procedures adhered to the Declaration of Helsinki.

### Participants

**FIGURE 1:**
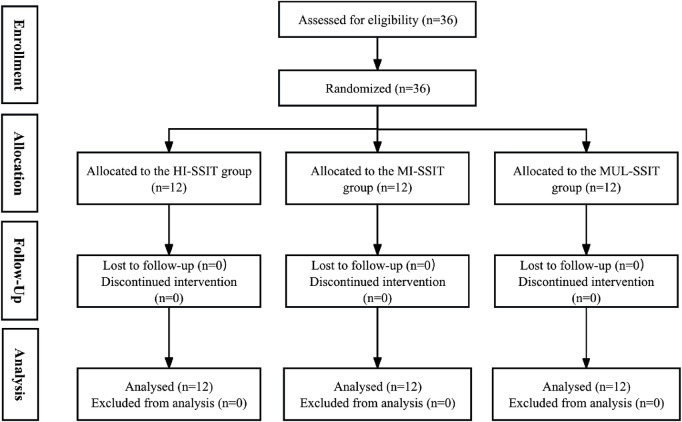
CONSORT flow diagram of participant allocation and analysis across three SSIT groups.

Sample size calculations were performed using G*Power software (version 3.1.9.7) based on previous research evaluating SSIT interventions in team sport athletes (Wilson et al., 2021), with an 80% power, α = 0.05, and an expected medium-to-large effect size (partial η^2^ = 0.14) for the primary outcomes. Thirty-six female basketball players (age: 20.7 ± 1.8 years; height: 177.3 ± 6.2 cm; weight: 70.4 ± 7.5 kg) from two collegiate teams volunteered for this study. Inclusion criteria were: 1 at least 3 years of competitive basketball experience, 2 participation in regular team training sessions (≥4 sessions/week), 3 no injuries in the previous 3 months, and 4 no contraindications to high-intensity exercise. Exclusion criteria included: 1 any cardiovascular or orthopedic conditions exacerbated by intense exercise, 2 inability to complete baseline testing, and 3 planned absence for more than three consecutive days during the intervention period. After baseline testing, participants were stratified by playing position (guards, forwards, centers) and randomly assigned to one of three training groups via a computer-generated randomization sequence: High-Intensity SSIT (HI-SSIT, n = 12), Moderate-Intensity SSIT (MI-SSIT, n = 12), and Multiple-Intensity SSIT (MUL-SSIT, n = 12). None of the participants had previous systematic experience with the SSIT prior to the intervention (Figure 1). To ensure familiarity with all testing procedures and minimize potential learning effects, participants completed a familiarization session 1 week before baseline testing.

### Procedures

#### Testing schedule

The study was conducted during the pre-season period to minimize the impact of competitive games on the training intervention. Testing sessions were completed at three time points:

Baseline (PRE): 1 week before the start of the intervention.

Post-intervention (POST): 1 week after completion of the 8-week intervention.

All testing sessions were conducted at the same time of day (±1 h) to minimize the effects of diurnal variation and were separated into two consecutive days. Day 1 included anthropometric measurements, vertical jump tests, sprint and agility tests, and the Yo-Yo Intermittent Recovery Test. Day 2 included skill performance tests and repeated sprint ability assessment.

#### Anthropometric measurements

Body mass was measured to the nearest 0.1 kg using a calibrated electronic scale (Seca 769, Hamburg, Germany), with participants wearing light clothing and no shoes. Standing height was measured to the nearest 0.1 cm using a stadiometer (Seca 217, Hamburg, Germany). Body composition was assessed using bioelectrical impedance analysis (InBody 770, Seoul, South Korea) following standardized procedures. The InBody 770 has demonstrated high test-retest reliability for body fat percentage measurements, with intraclass correlation coefficients (ICC) exceeding 0.95 (2013).

#### Vertical jump performance

Countermovement jump (CMJ) and approach jump performance were assessed using a portable force platform (Kistler 9286BA, Winterthur, Switzerland) sampling at 1,000 Hz. For the CMJ, participants stood with feet shoulder-width apart, hands on hips, and performed a countermovement to a self-selected depth followed by a maximal vertical jump (Di Domenico et al., 2023). For the approach jump, participants were allowed a 3-step approach before performing a maximal vertical jump, simulating a basketball-specific jumping task. Three attempts were performed for each jump type with 2-min rest intervals, and the highest jump height was recorded. Jump height was calculated from flight time using the equation: h = gt^2^/8, where h is the jump height (m), g is the acceleration due to gravity (9.81 m/s^2^), and t is the flight time (s) Additionally, a 30-s continuous jump test was performed to assess lower-limb muscular endurance and fatigue resistance. Participants performed repeated CMJs for 30 s, attempting to maximize jump height while minimizing ground contact time. The total number of jumps and the jump height decrement (%) from the first to the last five jumps were recorded. Multiple studies report excellent test-retest and intrasession reliability for CMJ height and related variables, with ICCs typically ranging from 0.79 to 0.98 and coefficients of variation (CV) generally below 10% (Heishman et al., 2020).

#### Sprint and agility performance

Linear sprint performance was assessed over 10 m and 20 m distances using photocell timing gates (Microgate Witty, Bolzano, Italy) positioned at the start, 10 m, and 20 m marks. Participants started in a stationary split stance with the front foot 0.5 m behind the first timing gate. Three attempts were performed with 3-min recovery periods, and the fastest time was recorded. Short-distance sprints (10–20 m), when measured with timing gates or similar precise technology, consistently show high test-retest reliability, with ICC values typically ranging from about 0.76 to 0.94 (Bariya and Pathak, 2020).

Change-of-direction ability was assessed using the modified T-test (Radhouane Haj Sassi et al., 2009). Four cones were arranged in a T-shape, with a 5 m distance from the start to the middle cone, and 2.5 m to each side cone. Participants sprinted forward to the middle cone, side-shuffled to the right cone, side-shuffled to the left cone, side-shuffled back to the middle cone, and backpedaled to the starting position. Three trials were performed with 3-min recovery periods, and the fastest time was recorded. Studies report ICCs for the modified T-test ranging from 0.82 to 0.95, indicating strong reliability across different populations, including healthy adults, athletes, and both men and women (Sassi et al., 2009).

Basketball-specific defensive movement was assessed using the defensive slide test. Participants performed defensive slides between cones placed 5 m apart in a zig-zag pattern for a total distance of 30 m. Time was recorded using photocell timing gates. This test has demonstrated good test-retest reliability in basketball players, with ICCs above 0.74–0.82 (Vučković et al., 2022). Although these tests reflect physical components relevant to basketball gameplay, such as lateral movement speed and change-of-direction ability, they do not directly assess technical basketball skills (e.g., ball handling, shooting accuracy). Therefore, in this study, agility performance is used as a proxy for basketball-related movement efficiency, rather than sport-specific skill execution.

#### Repeated sprint ability (RSA)

RSA was assessed using a 6 × 20 m shuttle sprint test (Pareja-Blanco et al., 2016). Participants completed six 20 m shuttle sprints (10 m out and back) with 20 s of passive recovery between sprints. Performance was measured using the total sprint time (sum of all six sprints) and the percentage decrement score calculated as: [(total sprint time ÷ best sprint time × 6) − 1] × 100. This RSA protocol has demonstrated good reliability, with ICCs for total sprint time and fatigue index ranging from 0.85 to 0.91.

#### Intermittent endurance capacity

The Yo-Yo Intermittent Recovery Test Level 1 (YYIR1) was used to assess intermittent endurance capacity (Narazaki et al., 2008) The test consisted of 2 × 20 m shuttle runs at progressively increasing speeds controlled by audio signals. Between each shuttle, participants had a 10-s active recovery period (2 × 5 m jog). The test was terminated when a participant failed to reach the finish line in time for two consecutive shuttles or due to volitional exhaustion. The total distance covered was recorded as the test result. The YYIR1 has excellent test-retest reliability in both male and female athletes, with ICC values typically ranging from 0.87 to 0.98 (Deprez et al., 2014).

#### Fatigue protocol

A basketball-specific fatigue protocol was used to induce fatigue before the post-fatigue skill assessments (Scanlan and Aaron, 2012). The Basketball Exercise Simulation Test (BEST) was modified to include four 5-min quarters with 2-min rest periods between quarters. The protocol included basketball-specific movements such as sprinting, defensive sliding, jumping, and directional changes at intensities simulating game demands. Heart rate was continuously monitored during the protocol using heart rate telemetry (Polar Team2 Pro, Kempele, Finland). Prior studies have reported this protocol as a valid and repeatable method to simulate game-induced fatigue, though specific ICC values are not always provided.

### Physiological measurements

#### Heart rate monitoring

Heart rate was continuously monitored during all training sessions using heart rate telemetry (Polar Team2 Pro, Kempele, Finland). Maximum heart rate (HRmax) was determined using the Yo-Yo Intermittent Recovery Test at baseline. Heart rate recovery was assessed by recording heart rate at 30 s and 60 s post-exercise during training sessions. Polar heart rate monitors have demonstrated excellent agreement with ECG-derived heart rates, with ICCs typically above 0.95 and measurement errors below 2%.

#### Blood lactate concentration

Capillary blood samples (5 μL) were collected from the fingertip and analyzed for blood lactate concentration using a portable lactate analyzer (Lactate Pro 2, Arkray, Japan). Samples were collected at rest, immediately after the fatigue protocol, and at 3-, 5-, and 10-min post-exercise to assess lactate clearance rate.

#### Intervention protocol

All participants continued their re-gular basketball training (4-5 sessions per week, approximately 90 min per session, excluding SSIT sessions). The Short Sprint Interval Training (SSIT) intervention was conducted three times per week on non-consecutive days (Monday, Wednesday, and Friday) for 8 weeks. Each session was supervised by certified strength and conditioning specialists and began with a standardized 15-min warm-up, which included dynamic stretching, joint mobilization, and basketball-specific movements. High-Intensity SSIT (HI-SSIT): Participants performed 2-3 sets of 6-8 sprints at 90%–95% of maximal heart rate (RPE 9-10). Each sprint lasted 15–20 s, with 15–20 s passive recovery between sprints and 3 min active recovery between sets. Training progression was achieved by increasing the number of sprints per set (from 6 to 8) and the number of sets (from 2 to 3) over the 8-week period (Table 1).

**TABLE 1:** Training protocol characteristics for high-, moderate-, and multiple-intensity SSIT groups.

Group	Intensity	Sprint duration	Recovery	Session structure	Progression
High-Intensity SSIT (HI-SSIT)	90%–95% of maximal heart rate (RPE 9–10)	15–20 s	15–20 s passive recovery between sprints; 3 min active recovery between sets	2–3 sets of 6–8 sprints per set, with 15–20 s passive recovery between each sprint and 3 min of active recovery between sets. Warm-up: 15-min dynamic stretching and mobilization	Increase the number of sprints per set (from 6 to 8) and the number of sets (from 2 to 3) over the 8 weeks
Moderate-Intensity SSIT (MI-SSIT)	80%–85% of maximal heart rate (RPE 7–8)	20–25 s	20–25 s passive recovery between sprints; 3 min active recovery between sets	3–4 sets of 8–10 sprints per set, with 20–25 s passive recovery between each sprint and 3 min active recovery between sets. Warm-up: 15-min dynamic stretching and mobilization	Increase the number of sprints per set (from 8 to 10) and the number of sets (from 3 to 4) over the 8 weeks
Multiple-Intensity SSIT (MUL-SSIT)	90%–95% of maximal heart rate (RPE 9–10)	15–20 s	15–20 s passive recovery between sprints; 3 min active recovery between sets	Monday: High-intensity protocol (90%–95% HRmax, RPE 9–10) Wednesday: Moderate-intensity protocol (80%–85% HRmax, RPE 7–8) Friday: Undulating intensity (85%–95% HRmax within each set)	Progressively increase the intensity within each session. Monday: 90%–95% HRmax; Friday: intensity undulates between 85% and 95% HRmax

Note. RPE, Rating of Perceived Exertion (0–10 scale); HRmax, maximal heart rate. Progression was applied weekly to increase training load through either sprint number, set number, or intensity. Multiple-intensity SSIT, included varied intensity sessions throughout the week (e.g., high, moderate, and undulating formats). All sessions included a 15-min standardized warm-up.

#### Statistical analysis

Data were analyzed using SPSS software (version 26.0, IBM Corp., Armonk, NY, USA). Normality of distribution was confirmed using the Shapiro-Wilk test. Descriptive statistics are presented as means and standard deviations (mean ± SD). A 2 × 3 mixed-model analysis of variance (ANOVA) with repeated measures was used to examine the effects of group (HI-SSIT, MI-SSIT, MUL-SSIT) and time (PRE, POST) on each dependent variable. Mauchly’s test was used to assess sphericity, and where the assumption was violated, Greenhouse-Geisser corrections were applied.

When significant main effects or interactions were detected, *post hoc* pairwise comparisons with Bonferroni adjustment were conducted to identify specific differences. Effect sizes were calculated using partial eta squared (η^2^p) for ANOVA effects and Cohen’s d for pairwise comparisons, with values of 0.01, 0.06, and 0.14 representing small, medium, and large effects for η^2^p, and values of 0.2, 0.5, and 0.8 representing small, medium, and large effects for Cohen’s d, respectively (Taylor and Harris, 2023). Qualitative probabilities of effects being beneficial or detrimental were calculated using the smallest worthwhile change (SWC), defined as 0.2 × inter-subject SD, better or worse effects were assessed qualitatively as follows: <1%, almost certainly not; 1%–5%, very unlikely; 5%–25%, unlikely; 25%–75%, possibly; 75%–95%, likely; 95%–99%, very likely; and >99%, almost certain. If the chances of obtaining beneficial/better or detrimental/worse were both >5%, the true difference was assessed as unclear (Hopkins et al., 2009; Marques et al., 2019). Statistical significance was set at p < 0.05. To enhance practical interpretability for coaches and practitioners, these descriptors reflect both the magnitude and certainty of observed effects. For instance, a “possibly beneficial” outcome suggests moderate probability of performance improvement with individual variability, whereas a “likely beneficial” result implies high confidence and practical applicability. This approach helps translate statistical outcomes into meaningful guidance for sport-specific training decisions.

## Results

### Vertical jump performance

**FIGURE 2:**
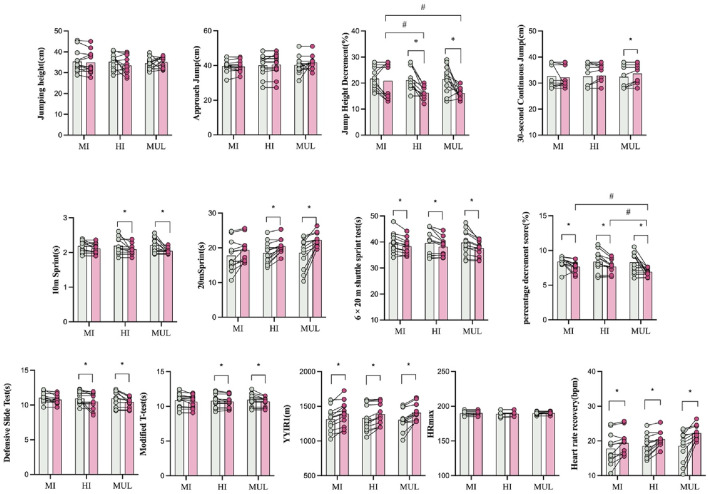
Pre- and post-intervention changes in six key performance variables, presented separately for each training group (HI-SSIT, MI-SSIT, and MUL-SSIT). Note: * indicates a significant within-group difference between pre- and post-intervention (p < 0.05); #indicates a significant between-group difference at post-test based on *post hoc*comparisons (p < 0.05).

For the countermovement jump (CMJ), there were no significant main effects of group (F = 0.326, p = 0.724), time (F = 1.467, p = 0.724), or group × time interaction (F = 1.405, p = 0.234). The MI-SSIT group demonstrated a small decline from 35.36 ± 5.36 cm to 34.93 ± 5.31 cm (Cohen’s d = 0.08), with a qualitative probability of “Unlikely” improvement. The HI-SSIT group showed a moderate decrease from 35.23 ± 3.89 cm to 33.39 ± 4.28 cm (Cohen’s d = −0.45), interpreted as a “Possibly” detrimental effect. The MUL-SSIT group showed a modest increase from 34.43 ± 2.81 cm to 35.12 ± 2.68 cm (Cohen’s d = 0.08), suggesting a “Possibly” beneficial outcome (Tables 2, 3; Figure 2).

**TABLE 2:** Pre- and post-intervention performance outcomes across groups.

Variable	High-intensity SSIT		Moderate-intensity		Multiple-intensity SSIT	
	Pre	Post	Pre	Post	Pre	Post
CMJ	35.23 ± 3.89	33.39 ± 4.28	35.36 ± 5.36	34.93 ± 5.31	34.43 ± 2.81	35.12 ± 2.68
Approach jump	40.04 ± 6.22	40.48 ± 6.41	39.69 ± 3.32	39.36 ± 3.25	40.27 ± 4.99	41.85 ± 3.90
Jump height decrement	21.08 ± 4.50	16.25 ± 2.34*#	21.67 ± 3.94	20.83 ± 7.03	21.50 ± 5.07	16.08 ± 2.15*#
30-secondcontinuousjump	32.58 ± 4.58	32.83 ± 4.09	32.67 ± 4.62	32.25 ± 3.96	32.42 ± 4.64	33.67 ± 3.68*
10 m Sprint	2.19 ± 0.23	2.09 ± 0.14*	2.18 ± 0.17	2.11 ± 0.14	2.21 ± 0.21	2.05 ± 0.07*
20 m Sprint	3.88 ± 0.23	3.79 ± 0.29*	3.77 ± 0.31	3.75 ± 0.32	3.74 ± 0.30	3.62 ± 0.31*
Modified T-test	10.87 ± 1.03	10.66 ± 0.92*	10.81 ± 0.97	10.70 ± 0.85	10.93 ± 0.92	10.70 ± 0.67*
Defensive slide test	10.97 ± 0.91	10.51 ± 1.16*	11.04 ± 0.72	10.77 ± 0.62	10.98 ± 1.13	10.43 ± 0.72*
6 × 20 m shuttle sprint test	39.59 ± 4.93	38.19 ± 3.44*	39.69 ± 3.79	38.34 ± 2.94*	39.73 ± 4.76	37.62 ± 2.98*
Percentage Decrement score	8.40 ± 1.58	7.66 ± 1.01*	8.28 ± 1.46	7.58 ± 0.80*#	8.41 ± 0.80	6.91 ± 0.56*#
YYIR1	1329.37 ± 189.39	1385.20 ± 158.40*	1311.14 ± 171.00	1392.85 ± 178.69*	1302.53 ± 148.89	1408.60 ± 120.83*
HRmax	188.75 ± 3.14	189.00 ± 2.86	189.92 ± 3.00	190.17 ± 2.79	189.92 ± 1.88	190.17 ± 1.90
Heart rate recovery	18.53 ± 3.14	20.47 ± 2.13*	17.80 ± 4.38	19.39 ± 3.37*	18.57 ± 4.38	22.23 ± 1.97*

Note. Values are presented as mean ± standard deviation (SD). *Indicates a statistically significant difference between pre- and post-intervention within the same group (*p < 0.05). # indicates a statistically significant difference between the MUL-SSIT, group and at least one of the other groups at post-test (p < 0.05). CMJ, countermovement jump; YYIR1 = Yo-Yo Intermittent Recovery Test Level 1; HRmax, maximal heart rate.

**TABLE 3:** Effect sizes and probabilities of performance changes following SSIT interventions.

Variable	High-intensity SSIT		Moderate-intensity		Multiple-intensity SSIT	
	Cohen’s d	Percent changes of Better/Trivial/Worse effect	Cohen’s d	Percent changes of Better/Trivial/Worse effect	Cohen’s d	Percent changes of Better/Trivial/Worse effect
CMJ	0.45	17/25/58 Possibly	0.08	25/25/50 unlikely	0.08	66.67/8.33/25 Possibly
Approach jump	0.07	25/33.33/41.67 unlikely	0.1	16.67/83.33/0 unlikely	0.1	41.67/50/8.33 Possibly
Jump height decrement	1.35	25/33.33/41.67 unlikely	0.15	0/25/75 most unlikely	0.15	25/0/75 unlikely
30-secondcontinuousjump	0.06	25/58.33/16.67 unlikely	0.1	25/66.67/8.33 unlikely	0.1	50/50/0 Possibly
10 m Sprint	0.54	16.67/41.67/41.67 unlikely	0.45	0/66.67/33.33 most unlikely	0.45	0/50/50 most unlikely
20 m Sprint	0.37	50/8.33/41.67 Possibly	0.08	50/0/50 Possibly	0.08	0/41.67/58.33 most unlikely
Modified T-test	0.21	16.67/50/33.33 unlikely	0.12	8.33/58.33/33.33 unlikely	0.12	0/58.33/41.67 most unlikely
Defensive slide test	0.44	16.67/25/58.33 unlikely	0.41	0/66.67/33.33 most unlikely	0.41	0/33.33/66.67 most unlikely
6 × 20 m shuttle sprint test	0.33	8.33/8.33/88.33 unlikely	0.4	16.67/25/58.33 unlikely	0.4	8.33/16.67/75 unlikely
Percentage Decrement score	0.55	8.33/16.67/75 unlikely	0.6	8.33/25/66.67 unlikely	0.6	25/16.67/58.33 unlikely
YYIR1	0.32	83.33/16.67/0 likely	0.47	58.33/41.67/0 possibly	0.47	91.67/8.33/0 likely
HRmax	0.08	16.67/83.33/0 unlikely	0.09	16.67/83.33/0 unlikely	0.09	16.67/75/8.33 unlikely
Heart rate recovery	0.72	58.33/33.33/8.33 possibly	0.41	83.33/0/16.67 likely	0.41	91.67/8.33/0 likely

Note. Cohen’s d represents the effect size between pre- and post-intervention. Percentages represent the likelihood of better, trivial, or worse outcomes based on magnitude-based inference. Interpretation: <1% = almost certainly not; 1%–5% = very unlikely; 5%–25% = unlikely; 25%–75% = possibly; 75%–95% = likely; 95%–99% = very likely; >99% = almost certainly. Performance outcomes are categorized as: CMJ, countermovement jump; RSA, repeated sprint ability; HR, heart rate.

For the approach jump, no significant main effects were found for group (F = 0.326, p = 0.724), time (F = 1.467, p = 0.234), or interaction (F = 1.405, p = 0.260). The HI-SSIT group showed a trivial increase from 40.04 ± 6.22 cm to 40.48 ± 6.41 cm (Cohen’s d = 0.07), rated as “Unlikely.” The MI-SSIT group slightly declined from 39.69 ± 3.32 cm to 39.36 ± 3.25 cm (Cohen’s d = 0.10), also “Unlikely.” The MUL-SSIT group improved from 40.27 ± 4.99 cm to 41.85 ± 3.90 cm (Cohen’s d = 0.10), corresponding to a “Possibly” beneficial effect (Tables 2, 3; Figure 2).

Jump height decrement showed a significant group × time interaction (F = 3.277, p = 0.050) and a significant main effect of time (F = 21.555, p < 0.001). The HI-SSIT group demonstrated a substantial reduction in decrement score from 21.08% ± 4.50% to 16.25% ± 2.34% (Cohen’s d = 1.35), indicating “Possibly” better fatigue resistance. The MUL-SSIT group also exhibited a notable improvement from 21.50% ± 5.07% to 16.08% ± 2.15% (Cohen’s d = 1.35), while the MI-SSIT group showed minimal change from 21.67% ± 3.94% to 20.83% ± 7.03% (Cohen’s d = 0.15), considered “Most unlikely” to be beneficial (Tables 2, 3; Figure 2).

The 30-s continuous jump test revealed no significant group effect (F = 0.056, p = 0.946) or group × time interaction (F = 0.231, p = 0.794). The HI-SSIT group remained nearly unchanged (32.83 ± 4.09 cm to 32.58 ± 4.58 cm, d = 0.06), with “Unlikely” improvement. The MI-SSIT group slightly improved (32.25 ± 3.96 cm to 32.67 ± 4.62 cm, d = 0.10), also “Unlikely.” The MUL-SSIT group improved from 32.42 ± 4.64 cm to 33.67 ± 3.68 cm (d = 0.10), with a probability of “Possibly” better performance (Tables 2, 3; Figure 2).

### Sprint and agility performance

For linear sprint performance, there were significant time effects in both 10 m (F = 16.584, p < 0.001) and 20 m sprints (F = 15.611, p < 0.001), with no significant group × time interaction. The HI-SSIT group improved from 2.19 ± 0.23 s to 2.09 ± 0.14 s in the 10 m sprint (Cohen’s d = 0.54) and from 3.88 ± 0.23 s to 3.79 ± 0.29 s in the 20 m sprint (d = 0.37), both showing “Possibly” beneficial effects. The MI-SSIT group also improved, from 2.18 ± 0.17 s to 2.11 ± 0.14 s (10 m, d = 0.45) and from 3.77 ± 0.31 s to 3.75 ± 0.32 s (20 m, d = 0.08), with qualitative effects rated as “Unlikely” to “Possibly.” The MUL-SSIT group showed the largest gain in 10 m sprint performance (2.21 ± 0.21 s to 2.05 ± 0.07 s, d = 1.07) and a moderate improvement in 20 m sprint (3.74 ± 0.30 s to 3.62 ± 0.31 s, d = 0.39), both rated as “Possibly” beneficial (Tables 2, 3; Figure 2).

For the Modified T-test, there were no significant effects for group (F = 0.577, p = 0.567) or group × time interaction (F = 0.017, p = 0.983), but a significant time effect (F = 14.898, p = 0.001) was found. All three groups showed marginal improvements, with HI-SSIT (10.87 ± 1.03 s to 10.66 ± 0.92 s), MI-SSIT (10.81 ± 0.97 s to 10.70 ± 0.85 s), and MUL-SSIT (10.93 ± 0.92 s to 10.70 ± 0.67 s), each associated with small effect sizes (d = 0.12–0.21) and qualitative probabilities rated as “Unlikely” or “Most unlikely (Tables 2, 3; Figure 2).”

The defensive slide test also showed no significant group (F = 0.277, p = 0.192) or interaction effect (F = 0.635, p = 0.536), although a significant time effect was observed (F = 17.878, p < 0.001). The HI-SSIT group improved from 10.97 ± 0.91 s to 10.51 ± 1.16 s (d = 0.44), MI-SSIT from 11.04 ± 0.72 s to 10.77 ± 0.62 s (d = 0.41), and MUL-SSIT from 10.98 ± 1.13 s to 10.43 ± 0.72 s (d = 0.41), but the improvements were generally rated as “Unlikely” or “Most unlikely (Tables 2, 3; Figure 2).”

### Repeated sprint ability (RSA)

The 6 × 20 m shuttle sprint test showed a significant main effect of time (F = 47.239, p < 0.001), with all three groups improving. The HI-SSIT group reduced total sprint time from 39.59 ± 4.93 s to 38.19 ± 3.44 s (d = 0.33), the MI-SSIT group from 39.69 ± 3.79 s to 38.34 ± 2.94 s (d = 0.40), and the MUL-SSIT group from 39.73 ± 4.76 s to 37.62 ± 2.98 s (d = 0.40). Despite improvements, qualitative probabilities were interpreted as “Unlikely” or borderline “Possibly” across groups. The percentage decrement score also showed a significant time effect (F = 32.398, p < 0.001). HI-SSIT improved from 8.40 ± 1.58 to 7.66 ± 1.01 (d = 0.55), MI-SSIT from 8.28 ± 1.46 to 7.58 ± 0.80 (d = 0.60), and MUL-SSIT from 8.41 ± 0.80 to 6.91 ± 0.56 (d = 0.60). All groups showed statistically meaningful reductions in fatigue, though qualitative probabilities were rated as “Unlikely (Tables 2, 3; Figure 2).”

### Intermittent endurance capacity

YYIR1 performance significantly improved across all groups (F = 73.265, p < 0.001), with no significant group (F = 0.793, p = 0.461) or group × time interaction (F = 0.000, p = 1). The HI-SSIT group increased from 1329.37 ± 189.39 m to 1385.20 ± 158.40 m (d = 0.32), the MI-SSIT group from 1311.14 ± 171.00 m to 1392.85 ± 178.69 m (d = 0.47), and the MUL-SSIT group from 1302.53 ± 148.89 m to 1408.60 ± 120.83 m (d = 0.47). These improvements were interpreted as “Likely” for HI-SSIT and MUL-SSIT and “Possibly” for MI-SSIT (Tables 2, 3; Figure 2).

### Heart rate recovery

There was a significant main effect of time (F = 40.506, p = 0.000), with no significant group (F = 0.972, p = 0.389) or interaction effect (F = 2.859, p = 0.072). Heart rate recovery improved in all groups: HI-SSIT from 18.53 ± 3.14 to 20.47 ± 2.13 bpm (d = 0.72), MI-SSIT from 17.80 ± 4.38 to 19.39 ± 3.37 bpm (d = 0.41), and MUL-SSIT from 18.57 ± 4.38 to 22.23 ± 1.97 bpm (d = 0.41). These were qualitatively interpreted as “Possibly” to “Likely” beneficial adaptations (Tables 2, 3; Figure 2).

## Discussion

The present study investigated the effects of multiple-intensity SSIT pairing patterns on explosive performance and skill performance in female basketball players. Our findings indicate that, while there were no significant overall improvements in vertical jump performance as measured by the CMJ and approach jump, specific aspects such as jump height decrement and fatigue resistance, as well as linear sprint and repeated sprint ability, demonstrated meaningful modulations following the intervention. These outcomes underscore the complex interplay between training intensity, neuromuscular adaptation, and sport-specific performance, and they contribute to an emerging body of literature seeking to optimize performance through tailored training protocols.

In our study, the CMJ performance did not reveal significant main effects of group or time. The heterogeneous responses in jump performance found in this study align with previous investigations that have utilized various field-based assessments to quantify lower-limb explosive performance. For example, Chamari et al. (2008) demonstrated that the five-jump test can effectively quantify explosive performance in soccer players (Chamari et al., 2008); similarly, Stockbrugger and Haennel’s validation of the medicine ball throw test (Stockbrugger and Haennel, 2001) supports the need for multiple testing modalities to capture the multifaceted nature of explosive performance. Moreover, Cronin et al. (2001) emphasized that movement technique plays a critical role in optimizing explosive performance output (Cronin et al., 2001). The divergent outcomes in jump height and fatigue resistance found here suggest that the pairing patterns used in SSIT may influence neuromuscular fatigue differently, perhaps by engaging distinct motor unit recruitment strategies. These observations resonate with prior work that noted varied fatigue responses in athletes under different training regimes (Rejc et al., 2018; Haj-Sassi et al., 2011). Such findings underscore the importance of monitoring not only peak performance but also the sustainability of performance under conditions of repeated explosive effort.

Significant enhancements in linear sprint performance were evident in both the 10 m and 20 m sprint tests across all groups, with the MUL-SSIT group displaying the most notable improvements in the 10 m sprint. Although the group × time interaction did not achieve statistical significance, the substantial reduction in sprint times, particularly in short, explosive efforts, indicates favorable neuromuscular adaptations. Rapid acceleration over short distances is pivotal in basketball for instigating fast breaks and defensive transitions. These results align with existing literature associating improved sprint performance with enhanced explosive neuromuscular function. For example, Tillin et al. (2010) illustrated that explosive power athletes surpassed untrained individuals in rapid force generation during sprints (Tillin et al., 2010); similarly, Tsao et al. (2022) underscored the significance of motor abilities, including explosive power, in identifying athletic talent (Tsao et al., 2022). Furthermore, Gisladottir et al. (2024) investigated the relationship between agility and sprint performance, with their results supporting the correlation between linear speed and change-of-direction capabilities (Gisladottir et al., 2024). Considering the constant and rapid speed and direction changes in basketball, these advancements in sprint metrics bear substantial practical implications for athletic performance.

In contrast, the Modified T-test and the defensive slide test—which are measures of agility and change-of-direction speed—showed only marginal improvements. Although the time effects were significant in these tests, the lack of notable group differences suggests that SSIT pairing patterns may have a limited impact on the specific neuromuscular adaptations required for agility. This finding is not entirely surprising, as agility performance is influenced by multiple factors, including coordination, balance, and decision-making, which may not be sufficiently challenged by SSIT protocols alone (Castagna et al., 2009; Skelton et al., 2002). The relatively small effect sizes observed here suggest that while the SSIT approach may induce improvements in sprint capacity, integrating specific agility drills may be necessary to achieve more marked enhancements in change-of-direction speed.

Although certain performance indicators, such as CMJ and agility tests, did not yield statistically significant group or interaction effects, several outcomes showed small-to-moderate effect sizes (e.g., Cohen’s d = 0.4–0.5). In trained populations with relatively small sample sizes, such magnitudes may still reflect meaningful physiological adaptations. For example, the MUL-SSIT group exhibited “possibly beneficial” trends in CMJ and defensive slide performance, indicating improved neuromuscular output or movement efficiency not fully captured by conventional statistical thresholds. This apparent discrepancy may be further explained by the principle of training specificity. While SSIT mimics the intermittent, high-intensity demands of basketball, it does not sufficiently engage the perceptual and cognitive components critical to agility performance. Agility and defensive effectiveness rely not only on physical capabilities like acceleration and direction change, but also on rapid visual processing, anticipation, and decision-making (Young et al., 2015)—elements not directly trained by SSIT alone. Therefore, the modest improvements observed may reflect the limited transfer of linear sprint-based training to complex, multidimensional game behaviors. Future interventions might consider integrating SSIT with reactive agility drills or opponent-based scenarios to better simulate the perceptual-motor demands of in-game movement. Additionally, the training duration, while sufficient to elicit improvements in other performance domains, may have been insufficient to induce the specific neuromuscular adaptations required for greater agility and defensive sliding gains.

The RSA test, which consisted of a 6 × 20 m shuttle sprint, demonstrated significant improvements over time across all groups, with reductions in total sprint time and percentage decrement scores. These improvements in RSA reflect a better ability to sustain high-intensity efforts over multiple sprints—a quality that is crucial for the demands of basketball, where players often engage in bursts of rapid movements interspersed with short recovery periods. The substantial reductions in fatigue, as measured by the decrement scores, suggest that the HI-SSIT and MUL-SSIT conditions may enhance neuromuscular endurance and delay the onset of fatigue. Such adaptations are consistent with previous studies that reported alterations in muscle fiber recruitment and metabolic efficiency following high-intensity training protocols (Rejc et al., 2018). Furthermore, studies employing blood flow restriction training have demonstrated that modifications in training intensity can yield improvements in explosive power and fatigue resistance (Wang et al., 2023). In tandem with improvements in the RSA test, intermittent endurance capacity, as measured through the YYIR1 test, was significantly enhanced in all groups. These findings are indicative of improved aerobic capacity, which is essential for maintaining performance during prolonged, high-intensity activity. The aerobic and anaerobic systems must synergistically support explosive actions in basketball, and our results suggest that SSIT protocols can positively affect both energy systems (Erčulj et al., 2010). The concomitant improvement in heart rate recovery further supports the notion that SSIT fosters favorable cardiovascular adaptations (Ekstrand et al., 2013).

The performance enhancements observed in the study are attributed to neural and metabolic adaptations resulting from the specific pairing patterns utilized in the SSIT protocols. Differentiation between HI-SSIT, MI-SSIT, and MUL-SSIT is believed to influence motor unit recruitment patterns, rate of force development, and muscle fiber type utilization. Research by Zhang et al. (2023) suggests that resistance training methods, whether velocity-based or percentage-based, can have varying effects on explosive neuromuscular adaptations (Zhang et al., 2023). Additionally, findings by Fu et al. (2023) demonstrate acute potentiation effects following flywheel training, shedding light on how specific loading patterns can enhance explosive performance (Fu et al., 2023). Moreover, Zamparo et al. (1997) discuss the role of elastic recoil in enhancing muscle power, providing a potential biomechanical mechanism that may interact with the SSIT protocols (Zamparo et al., 1997).

The practical implications of this study are significant for practitioners seeking to optimize training regimens for female basketball players. The differential responses observed across SSIT intensities suggest that a multiple-intensity approach may be more beneficial in simultaneously enhancing neuromuscular endurance, speed, and explosive power compared with a single-intensity approach. This notion is supported by previous research reporting that individualized or mixed training modalities can yield superior performance benefits (Shalom et al., 2024; Crow et al., 2012). Coaches and practitioners might consider incorporating both high- and moderate-intensity elements into training sessions, with particular attention to fatigue resistance and sprint performance, which were among the most responsive outcomes.

Despite promising findings, several limitations must be acknowledged. First, the relatively small sample size (n = 12 per group) and the specific demographic—female collegiate basketball players from China—limit the generalizability of the findings. Future research should include larger and more diverse populations to validate and refine dosing recommendations across broader athletic cohorts. Second, although a comprehensive performance test battery was employed (including vertical and approach jump assessments, sprint tests, and repeated sprint ability measures), the ecological validity of these assessments may be limited compared to in-game performance indicators. Prior literature has highlighted the importance of field-based measures that closely simulate competitive conditions. Third, individual biomechanical and anatomical differences—such as variations in foot arch structure—may have influenced training responses, as suggested by previous studies, and warrant further investigation. Lastly, potential confounding variables, including hormonal status (e.g., menstrual cycle phase), nutritional intake, and recovery strategies, were not systematically monitored or controlled. These factors could have variably impacted performance outcomes and should be addressed in future studies.

## Conclusion

In conclusion, our study shows that various SSIT pairing patterns at different intensities lead to distinct improvements in explosive power, sprint performance, and fatigue resistance among female basketball players. These findings suggest the importance of incorporating mixed-intensity training methods to address the multifaceted physical demands of basketball. Future research should aim for larger-scale investigations, improved field-based evaluations, and personalized training strategies to better connect theoretical results with real-world basketball performance.

## Data Availability

The original contributions presented in the study are included in the article/supplementary material, further inquiries can be directed to the corresponding authors.
